# Pilot Study on the Use of DNA Priming Immunization to Enhance *Y. pestis* LcrV-Specific B Cell Responses Elicited by a Recombinant LcrV Protein Vaccine

**DOI:** 10.3390/vaccines2010036

**Published:** 2013-12-27

**Authors:** Wei Li, Shixia Wang, Shan Lu

**Affiliations:** Department of Medicine, University of Massachusetts Medical School, Worcester, MA 01605, USA; E-Mails: wei.li@umassmed.edu (W.L.); shixia.wang@umassmed.edu (S.W.)

**Keywords:** *Yersinia pestis*, V antigen, DNA vaccine, memory B cell

## Abstract

Recent studies indicate that DNA immunization is powerful in eliciting antigen-specific antibody responses in both animal and human studies. However, there is limited information on the mechanism of this effect. In particular, it is not known whether DNA immunization can also enhance the development of antigen-specific B cell development. In this report, a pilot study was conducted using plague LcrV immunogen as a model system to determine whether DNA immunization is able to enhance LcrV-specific B cell development in mice. Plague is an acute and often fatal infectious disease caused by *Yersinia pestis* (*Y. pestis*). Humoral immune responses provide critical protective immunity against plague. Previously, we demonstrated that a DNA vaccine expressing LcrV antigen can protect mice from lethal mucosal challenge. In the current study, we further evaluated whether the use of a DNA priming immunization is able to enhance the immunogenicity of a recombinant LcrV protein vaccine, and in particular, the development of LcrV-specific B cells. Our data indicate that DNA immunization was able to elicit high-level LcrV antibody responses when used alone or as part of a prime-boost immunization approach. Most significantly, DNA immunization was also able to increase the levels of LcrV-specific B cell development. The finding that DNA immunization can enhance antigen-specific B cell responses is highly significant and will help guide similar studies in other model antigen systems.

## 1. Introduction

DNA immunization was discovered about 20 years ago. While it was initially considered a novel approach to elicit T cell responses, data accumulated in the last decade has further indicated that DNA immunization is also very effective in eliciting antibody responses against both viral and bacterial antigens [1,2,3,4,5,6,7], particularly when included as part of a prime-boost immunization [8]. 

However, the mechanism by which DNA vaccines elicit high quality antibody responses has not been well studied. One possibility is that DNA immunization can elicit high quality antigen-specific B cell responses. In the current report, a pilot study was conducted to address this possibility using the LcrV (V antigen) from *Y. pestis* as a model antigen. *Y. pestis* is a gram-negative bacterium that causes human plague, which may present as one of three forms: bubonic, septicemic, or pneumonic, depending on the route of initial infection. Regardless of the route of infection, the disease results in high mortality (50%–90%) if left untreated [9]. An interest in a prophylactic vaccine against plague extends beyond biodefense applications, as isolated plague outbreaks occur sporadically in both developed and developing countries, and antibiotic-resistant strains have been described [10,11,12]. Currently, there is no widely acceptable vaccine against plague. Live attenuated strains and, more recently, formalin-killed whole cell vaccines have been developed but proved highly reactogenic in humans [13,14]. A killed whole-cell vaccine was licensed in the U.S. but was withdrawn from clinical use because it required multiple doses, was highly reactogenic, and did not protect effectively against pneumonic plague [13,14]. The F1 capsular protein (F1) and the V protein (LcrV, a component of the *Y. pestis* type-III secretion system) have been established as lead antigens for subunit-based plague vaccines and were shown to induce protection against bubonic and pneumonic plague in several animal models [5,7,14,15,16,17,18,19]. These antigens also elicited antibodies when administered in humans, however, the antibody response levels were moderate [20]. 

Our previous mouse studies established the feasibility of using DNA immunization to elicit LcrV antibody responses; mice immunized with LcrV DNA vaccines were protected from lethal mucosal challenges [5]. In the current study, the same LcrV DNA vaccines were used. Given mounting evidence from both plague and non-plague vaccines studies showing that protective immunity can be significantly improved when vaccines in different forms are administered in a prime-boost format [21,22,23,24,25,26], both DNA vaccine alone and DNA prime-protein boost approaches were included in the current study. We tested whether the heterologous DNA prime-protein boost approach is more effective than the homologous DNA alone or protein alone immunization approaches in eliciting LcrV antigen-specific B cell immune responses.

## 2. Experimental

### 2.1. LcrV DNA Vaccine

The codon optimized DNA vaccine (V.opt) expressing the LcrV protein of *Y. pestis* was constructed, as previously described [27]. A synthetic *lcrV* gene was cloned into the DNA vaccine vector, pSW3891 [26], at the *Pst*I and *BamH*I sites downstream of the cytomegalovirus (CMV) immediate early (IE) promoter and its adjacent Intron A [28,29]. The DNA plasmids used in this study were prepared by a Mega purification kit (QIAGEN).

### 2.2. V protein Vaccine

The wild type *lcrV* gene was PCR-amplified from the *LcrV* DNA vaccine, as previously described [5] and cloned into the *E. coli* expression vector, pBAD/gIII (Invitrogen), with a His(6)-Tag at the *C*-terminus fused with V-antigen. The plasmid DNA was transformed into *E. coli* strain, LMG194, for V antigen expression. LMG194 bacterial culture and protein expression were conducted following instructions from the pBAD/gIII kit from Invitrogen. The LcrV-His(x6) protein was purified from the LcrV expressing LMG194 bacterial lysate using a nickel column. The purified V protein was analyzed by SDS-PAGE and Western blot and used for V protein vaccination and ELISA to detect V-specific antibody responses in mouse sera. 

### 2.3. Mouse Immunization

**Figure 1:**
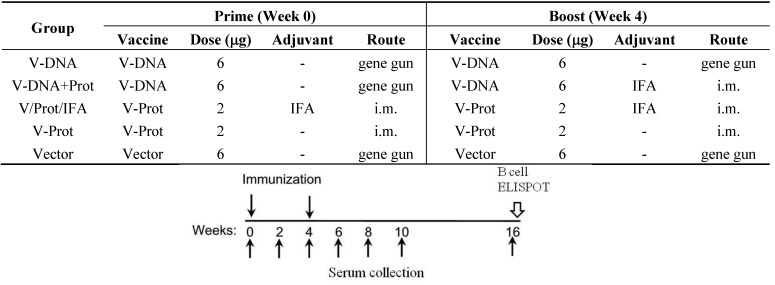
*lcrV*-DNA and V-protein immunization groups and vaccine components. Each mouse received 2 immunizations: prime at Week 0 and boost at Week 4 using designated codon optimized V DNA vaccine (V-DNA), V protein (V-Prot), or empty DNA vaccine vector (Vector) as indicated.

Female BALB/c mice of 6–8 weeks old were purchased from Taconic Farms (Germantown, NY, USA) and housed in the animal facility managed by the Department of Animal Medicine at the University of Massachusetts Medical School (UMMS) in accordance with IACUC approved protocol. Mice (5/group) received two immunizations at Weeks 0 and 4 with designated vaccination regimens listed in Figure 1. Each mouse received codon optimized *lcrV* DNA vaccine (V-opt) (X2), V protein alone (X2), V-protein formulated with Incomplete Freund Adjuvant (IFA) (X2), V-opt DNA prime followed by V protein/IFA boost, or DNA vector alone immunization as the negative control. DNA immunizations were conducted via gene gun using a Helios gene gun (Bio-Rad). V.opt or the pSW3891 vector plasmid was coated onto 1.0-micron gold beads at 2 μg DNA/mg gold. Each shot delivered 1 μg of DNA and a total of six non-overlapping shots were delivered to shaved abdominal skin at each immunization after animals were anesthetized. Protein immunizations were done by intramuscular (i.m.) injection at the quadriceps, one injection site at one leg each with a dose of 1 μg/site (X2 sites). Sera were collected prior to and at two weeks after each immunization and at additional time points as indicated in Figure 1. At Week 16, animals were euthanized and splenocytes and bone marrow cells were isolated for B cell assays.

### 2.4. ELISA (Enzyme-Linked Immunosorbent Assay)

Mouse sera were tested for V-specific IgG antibody responses by ELISA as previously described [5]. Microtiter plates were coated with 100 ng/well of purified recombinant V antigen (1 μg/mL in PBS, pH 7.2) at 4 °C overnight and then washed five times with washing buffer (PBS at pH 7.2 with 0.1% Triton X-100). Blocking was done with 200 µL/well of 4% milk-whey blocking buffer for 1 h at room temperature. After removal of the blocking buffer and another five washes, 100 μL of serially diluted mouse sera was added and incubated for 1 h. The plates were washed five times and incubated with 100 μL of biotinylated anti-mouse IgG (Vector Laboratories, Burlingame, CA, USA) diluted at 1:1,000 for 1 h followed with washes. Horseradish peroxidase-conjugated streptavidin (Vector Laboratories, Burlingame, CA, USA), diluted at 1:2,000, was added (100 μL/well) and incubated for 1 h. After the final set of washes, 100 μL of fresh TMB substrate (Sigma Aldrich, St. Louis, MO, USA) was added to each well and incubated for 3.5 min. The reaction was stopped by adding 25 μL of 2 M H_2_SO_4_, and the plate was measured at OD 450 nm. The temporal serum antibody response trend was monitored directly by OD value from ELISA and antibody titers at the peak response or selected time points were calculated based on the end titration of serum dilution of immune sera (>2 fold over the control sera).

For V-specific IgG isotype analysis, a modified ELISA was conducted as previously described [5]. Serially diluted commercial mouse IgG, IgG1, or IgG2a (Southern Biotech, Birmingham, AL, USA) were coated onto ELISA plates to establish individual standard curves. ELISA, as described above, was performed on the same plates and concentrations of V-specific mouse IgG, IgG1, or IgG2a were calculated according to the standard curves.

### 2.5. B cell ELISPOT for Antibody Secreting Cells (ASC) and Memory B Cells

After isolation of splenocytes and bone marrow cells, they were divided into three portions for different assays: (1) freshly isolated cells were used to measure V-specific ASC; (2) V protein stimulated cells were used to detect memory B cells; and (3) splenocytes or bone marrow cells cultured for 5 days without stimulation were used to detect long lasting ASC. For B cell stimulation, bulk splenocytes or bone marrow cells at 5 million cells/ml were stimulated with V protein (5 µg/mL) + IL-2 (20 units/mL) for 5 days before being used for ELISPOT assays. 

To conduct V-antigen-specific ELISPOT assays, MultiScreenHTS Filter ELISPOT Plates (Millipore, Billerica, MA, USA) were first coated with V antigen at a concentration of 2 μg/mL in PBS at 4 °C overnight, then blocked as described above. Freshly isolated, stimulated or cultured splenocytes or bone marrow cells (100 μL/well, 500,000 cells/well) in R10 medium with 0.1% β-ME were incubated in duplicate wells for 4 h at 37 °C. The plates were then washed and incubated with 100 μL of biotinylated goat-anti-mouse IgG diluted at 1:1,000 in dilution buffer from the ELISPOT kit above for 1 h. After additional washes, 100 μL of AP-conjugated streptavidin complex diluted at 1:2,000 in dilution buffer was added to each well for 1h at 37 °C. The plates were washed, and spots were developed after a 7 min color reaction using 1-STEP NBT/BCIP. IgG spot-forming cells (SFC) were counted. The results were expressed as the number of SFC per 10^6^ input cells. 

### 2.6. Statistical Analysis

Student’s *t*-test was performed to evaluate differences in V-specific antibody and B cell ELISPOT data between any two groups. 

## 3. Results

The current pilot study was conducted, using a mouse model, to determine the effects of DNA immunization on the development of LcrV-specific B cells. Groups of BALB/c mice (5 mice/group) were immunized using one of the following regimens (Figure 1): (1) DNA Alone Group—received two codon optimized LcrV (V-opt) DNA [27] immunizations via gene gun at Weeks 0 and 4; (2) Prime-Boost Group—received LcrV DNA vaccine at Week 0 and the recombinant LcrV protein formulated with adjuvant IFA at Week 4 by i.m. immunization; (3) Protein Alone Group—received LcrV protein with IFA twice at Weeks 0 and 4 without DNA priming immunization; (4) Protein No Adjuvant Group—was similar to the third group except that recombinant LcrV proteins were used alone without any adjuvant; (5) Negative Control Group—received an empty DNA vector without *lcrV* gene insert. Serum samples were collected prior to the start of immunization and every 2 weeks afterwards until Week 10; samples were then tested for levels of LcrV-specific antibody responses. Animals were terminated at Week 16, three months (12 weeks) after the last immunization and serum samples collected at that time were used to measure the persistence of antibody responses. Spleen and bone marrow were collected for the measurement of LcrV-specific antibody secreting cells (ASC).

**Figure 2:**
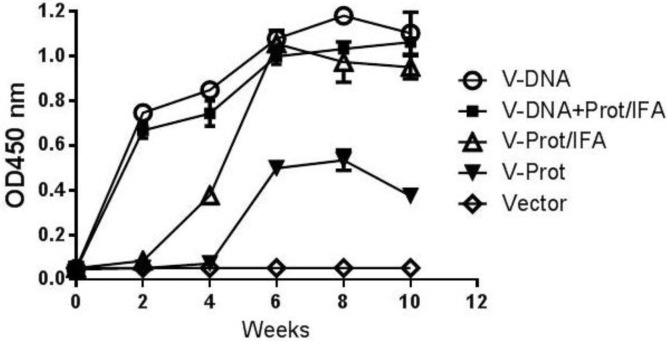
V-specific temporal antibody responses in mice immunized with different vaccination regimens: codon optimized V DNA vaccine alone (V-DNA), V DNA vaccine prime followed by V protein boost formulated with IFA (V-DNA+Prot/IFA), V protein formulated with IFA (V-Prot/IFA), V protein alone (V-Prot), or empty DNA vaccine vector alone (Vector). V-specific antibody responses were measured by ELISA at different time points using pooled mouse sera from each group against V protein. Each curve represents mean OD values with standard error of duplicated assays for each mouse group, at 1:500 serum dilution.

Gene gun delivery of the LcrV DNA vaccine was highly effective and positive antibody responses in the DNA Alone Group were detected even after a single immunization (Figure 2). The 2nd immunization was able to further increase antibody response levels, which continued to increase for about four weeks. The temporal antibody response pattern in the Prime-Boost Group was similar. In contrast, the Protein Alone Group had delayed and lower level antibody responses after one immunization, but was able to reach the same levels as those in the first two groups after the 2nd immunization. Adjuvant is important for the immunogenicity of LcrV protein vaccines as the LcrV protein alone without IFA (Protein No Adjuvant Group) was not able to elicit the same levels of antibody responses, even after two immunizations. There was no LcrV antibody response in the Negative Control Group. 

**Figure 3:**
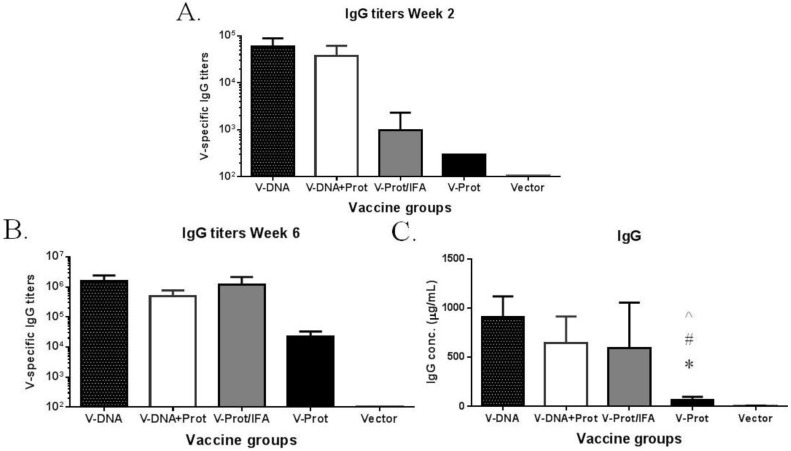
V-specific IgG responses induced by various V vaccine regimens in mice: codon optimized V DNA vaccine alone (V-DNA), V DNA vaccine prime followed by V protein boost formulated with IFA (V-DNA + Prot/IFA), V protein formulated with IFA (V-Prot/IFA), V protein alone (V-Prot), or empty DNA vaccine vector alone (Vector). Panels A and B: V-specific antibody titers were measured by ELISA against V protein in mouse sera collected at 2 weeks after the prime (1st immunization) (Panel A) or at 2 weeks after the boost (2nd immunization) (Panel B). Panel C: V-specific IgG concentrations at 2 weeks after the boost (2nd) immunization. Each bar represents the mean V-specific IgG titers or concentrations in each group of 5 mice with standard error. Statistically significant differences (*p < 0.05*) are indicated as “*”, “#” or “^” when comparing V-DNA, V-DNA + Prot/IFA, and V-Prot/IFA groups.

The temporal pattern of antibody responses was measured using a fixed serum dilution; therefore, additional ELISA studies were conducted to measure actual antibody titers (Figure 3). These results further confirm the temporal pattern using fixed serum dilutions. At Week 2, after the first immunization, antibody titers in the DNA primed groups were significantly higher than those in the two groups that received a protein vaccine as the prime (Figure 3A). By Week 6, which was two weeks after the 2nd immunization, LcrV-specific antibody titers in the DNA immunization groups were similar to those in the IFA-formulated recombinant LcrV protein group, but were still much higher than those in the group that received LcrV protein alone without IFA (Figure 3B). The same pattern was observed when the actual amount of LcrV-specific IgG (in μg/mL) was measured (Figure 3C) in addition to traditional end titration titers.

**Figure 4:**
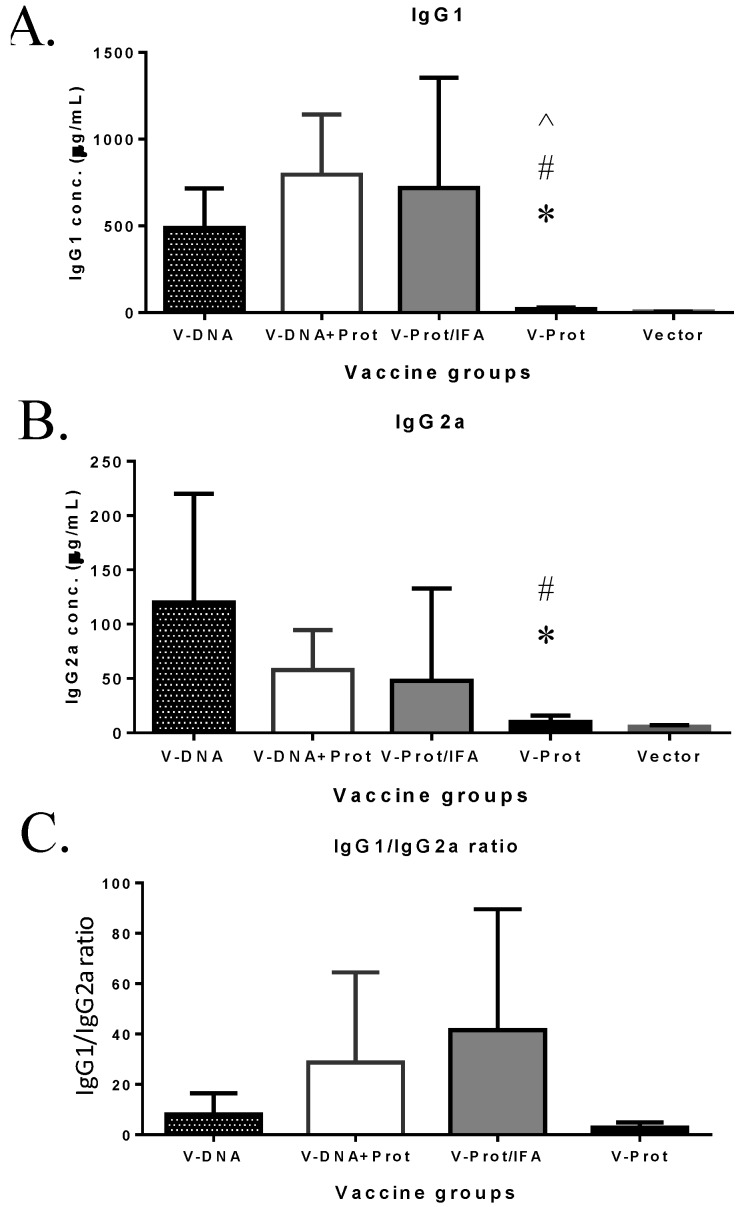
V-specific IgG1 and IgG2a responses induced by various V vaccine regimens in mice: codon optimized V DNA vaccine alone (V-DNA), V DNA vaccine prime followed by V protein boost formulated with IFA (V-DNA + Prot/IFA), V protein formulated with IFA (V-Prot/IFA), V protein alone (V-Prot), or empty DNA vaccine vector alone (Vector). V-specific IgG1 (Panel A) and IgG2a (Panel B) concentrations were measured by ELISA against V protein in mouse sera collected at 2 weeks after the boost (2nd) immunization. Each bar represents the mean IgG concentrations in each group of 5 mice with standard error. Statistically significant differences (*p <* 0.05) are indicated as “*”, “#” or “^” when comparing V-DNA, V-DNA + Prot/IFA, and V-Prot/IFA groups. Panel C: IgG1/IgG2a ratios determined at 2 weeks after the boost immunization. Each bar represents the mean IgG1/IgG2a ratios in each group of 5 mice with standard error.

Subtypes of IgG antibody responses were also measured (Figure 4). Regardless of the type of LcrV vaccine used, IgG1 levels (Figure 4A) were higher than IgG2a (Figure 4B), indicating a Th-2 type antibody dominated response with approaches used in the current study, which is consistent with the use of gene gun or adjuvant IFA, as reported in the literature [6,30]. However, recombinant LcrV protein with adjuvant IFA elicited a higher ratio of IgG1/IgG2a than that observed for the DNA vaccine groups (Figure 4C). Similar to total IgG responses, both IgG1 and IgG2a antibodies were significantly lower in the Protein No Adjuvant Group (Figure 4C). 

**Figure 5:**
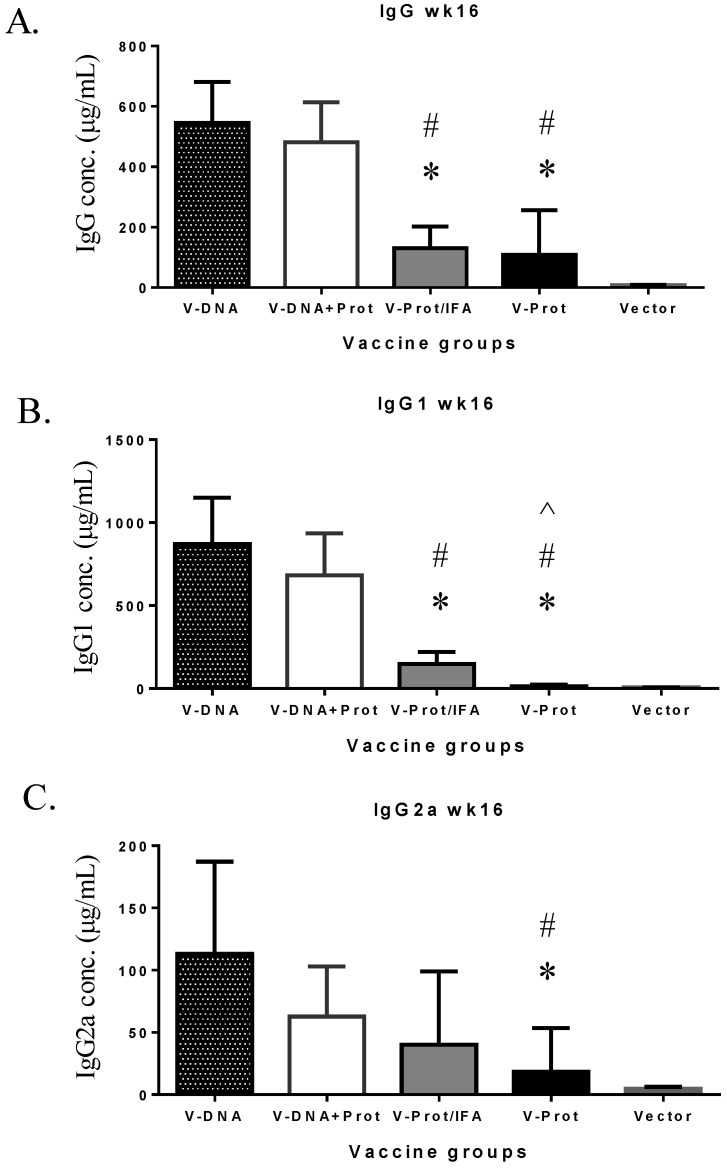
V-specific IgG (Panel A), IgG1 (Panel B), or IgG2a (Panel C) concentrations measured at 12 weeks after the boost immunization in mice receiving various V vaccine regimens: codon optimized V DNA vaccine alone (V-DNA), V DNA vaccine prime followed by V protein boost formulated with IFA (V-DNA + Prot/IFA), V protein formulated with IFA (V-Prot/IFA), V protein alone (V-Prot), or empty DNA vaccine vector alone (Vector). Antibody titers were measured by ELISA against V protein. Each bar represents the mean IgG concentration in each group of 5 mice with standard error. Statistically significant differences (*p <* 0.05) are indicated as “*”, “#” or “^” when comparing V-DNA, V-DNA + Prot/IFA, and V-Prot/IFA groups.

However, the longevity of LcrV antibody responses in the DNA vaccine primed groups was better maintained than that in the LcrV protein groups (Figure 5). At Week 16, or 12 weeks after the second immunization, total IgG (Figure 5A) and IgG1 (Figure 5B) levels were significantly lower in the two protein groups than in the two DNA primed groups. IgG2a levels in the two protein groups were also lower but the difference was only significant in the Protein No Adjuvant Group (Figure 5C).

**Figure 6:**
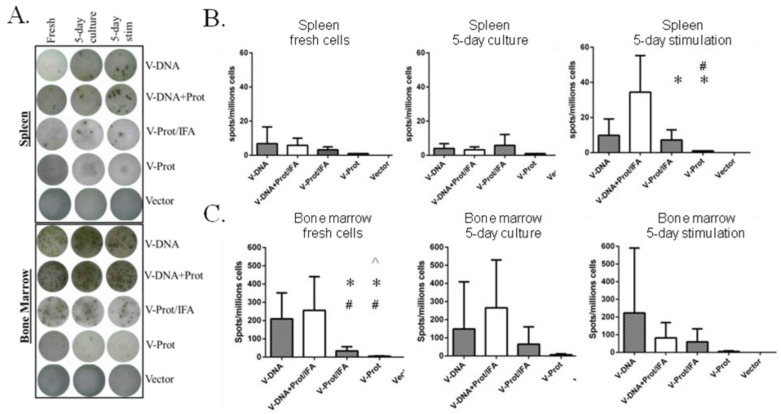
V-specific antibody secreting cells (ASC) in fresh, 5-day cultured and 5-day stimulated splenocytes and bone marrow cells as measured by ELISPOT. Mice immunized with different V vaccine regimens in mice: codon optimized V DNA vaccine alone (V-DNA),V DNA vaccine prime followed by V protein boost formulated with IFA (V-DNA + Prot/IFA), V protein formulated with IFA (V-Prot/IFA), V protein alone (V-Prot), or empty DNA vaccine vector alone (Vector), as indicated. Panel A: Actual sample wells of V-specific ASC spots splenocytes (upper panel) or bone marrow cells (lower panel). Panel B: Frequency of V-specific ASC per million splenocytes in each group. Panel C: Frequency of V-specific ASC per million bone marrow cells in each group. Data represent the mean spot forming cells (SFCs)/million cells with standard deviation from 5 mice/group. The splenocytes and bone marrow cells were collected 12 weeks after the boost (2nd) immunization. Statistically significant differences (*p <* 0.05) are indicated as “*”, “#” or “^” when comparing V-DNA, V-DNA + Prot/IFA, and V-Prot/IFA groups.

B cell ELISPOT analysis was conducted to measure LcrV-specific antibody secreting cells in the bone marrow and spleen of immunized mice. Fresh cells, cells cultured for five days without stimulation, and cells stimulated with LcrV antigen were used in this analysis (Figure 6). Figure 6A shows the representative ELISPOT pictures, which indicate that the overall frequency of LcrV-specific B cells in bone marrow was higher than that in the spleen. The recombinant LcrV protein vaccine alone group without adjuvant had the lowest levels of LcrV B cell responses in both bone marrow and spleen, which is compatible with observed antibody responses. When group average data was analyzed, the DNA prime-protein boost group had the highest LcrV B cell responses in spleen after stimulation, responses that were much higher than observed in the two LcrV protein groups (Figure 6B). In bone marrow, DNA vaccine groups generally had a higher number of LcrV B cells than protein vaccine groups, especially in fresh cells. However, due to the high variation in the B cell ELISPOT analysis in bone marrow cells, there was no statistically significant difference between DNA vaccine groups and protein groups in cultured or stimulated cells (Figure 6C).

## 4. Discussion

Historically, recombinant V protein immunization was shown effective in eliciting V-specific antibody responses and such antibody responses were responsible for the protective immunity in animal models [15,16,31]. A similar approach has advanced to human studies [20]. At the same time, other novel vaccination approaches have been reported in early preclinical studies. Our previous studies demonstrated that LcrV DNA vaccines could elicit significant levels of both LcrV-specific antibodies and CD8 T cell responses as part of the protective immunity against lethal intranasal *Y. pestis* challenge in a Balb/C mouse model [5,7,27]. 

Recent studies have suggested that a heterologous prime-boost vaccination approach, in which the same antigen is delivered sequentially by different types of vaccines, may be more effective in eliciting enhanced immune responses than the homologous prime-boost using the same type of vaccines [21]. We previously reported that DNA vaccine prime-protein or inactivated vaccine boost could significantly improve the overall immunogenicity and functional antibody responses against HIV [3,25] and influenza [22]. In the current study, we evaluated LcrV-specific antibody responses using the DNA prime-protein boost regimen based on our previous LcrV DNA vaccine work [3,5,7,25,27] with a new focus on the quality of antibody responses and, more importantly, the development of LcrV-specific B cells. 

A comparison of immunizations was conducted in this pilot mouse study with LcrV DNA alone, LcrV protein alone, or a combination of DNA prime-protein boost. We demonstrated that all regimens studied were capable of eliciting LcrV-specific antibody and B cell responses. However, the mice that received the LcrV DNA vaccine prime not only produced overall higher antibody titers but also generated improved B cells responses as measured by ELISPOT for the detection of LcrV-specific antibody secreting cells in fresh or stimulated splenocytes and bone marrow cells. 

Previous analysis of antibody isotypes suggested that a Th1-type (IgG2a dominant) antibody response may be important in providing better protection [5,27]. In addition to the levels of humoral responses, the mice that received the DNA prime produced more balanced IgG1/IgG2a responses with an increased level of IgG2a titers compared to the mice that received the protein alone immunization. These data highlight the potential of heterologous DNA prime-protein boosts to lead to higher Th1-type responses, which may be beneficial for protective immunity against plague. Besides antibody responses, our previous studies demonstrated that the V DNA vaccine could also induce antigen-specific T cell responses [7] similar to what reported by using other types of plague antigens to elicit T cell immune responses [32]. 

Another important finding from our study is that the levels of LcrV antibody responses were better maintained in the DNA prime-protein boost group compared to the other groups. This may imply that such a combination immunization approach may be more effective in building antigen-specific memory. To support this finding, the prime-boost approach was more effective in eliciting LcrV-specific B cell responses. It is interesting to observe that the benefit of the DNA prime is shown in the bone marrow with fresh cells, indicating the levels of available LcrV-specific antibody secreting cells, and in spleen with stimulated cells, indicating the levels of LcrV-specific memory B cells. These similar patterns of LcrV-specific B cell responses at different stages of development provided exciting evidence to support the use of DNA immunization to elicit high quality antibody and B cell responses. 

In summary, results from this study demonstrated that a heterologous DNA prime-protein boost approach elicits an improved antibody response and antigen-specific B cell responses against plague. This finding will have a major impact not only to the development of improved plague vaccines but also to the development of vaccines against other infectious-causing agents that require long term protection and high levels of antigen-specific memory B cell responses. Previously, there was limited information on the ability of DNA immunization to elicit high quality B cell responses. Information presented in this pilot study is highly significant and will set the stage for more in-depth studies with other antigen systems to fully establish this previously unknown mechanism of DNA vaccination.

## 5. Conclusions

The current report confirms previous findings that DNA immunization is effective in eliciting LcrV-specific antibody responses in a mouse model and further demonstrates that the DNA prime-protein boost approach is also effective in eliciting LcrV-specific B cell development, which is important for the quality and longevity of protective immunity. 
